# Understanding the fluid mechanics behind transverse wall shear stress

**DOI:** 10.1016/j.jbiomech.2016.11.035

**Published:** 2017-01-04

**Authors:** Yumnah Mohamied, Spencer J. Sherwin, Peter D. Weinberg

**Affiliations:** a Department of Aeronautics, Imperial College London, United Kingdom; b Department of Bioengineering, Imperial College London, United Kingdom

**Keywords:** Hemodynamics, Wall shear stress, Atherosclerosis, TransWSS, Planarity, Pulsatility

## Abstract

The patchy distribution of atherosclerosis within arteries is widely attributed to local variation in haemodynamic wall shear stress (WSS). A recently-introduced metric, the transverse wall shear stress (transWSS), which is the average over the cardiac cycle of WSS components perpendicular to the temporal mean WSS vector, correlates particularly well with the pattern of lesions around aortic branch ostia. Here we use numerical methods to investigate the nature of the arterial flows captured by transWSS and the sensitivity of transWSS to inflow waveform and aortic geometry. TransWSS developed chiefly in the acceleration, peak systolic and deceleration phases of the cardiac cycle; the reverse flow phase was too short, and WSS in diastole was too low, for these periods to have a significant influence. Most of the spatial variation in transWSS arose from variation in the angle by which instantaneous WSS vectors deviated from the mean WSS vector rather than from variation in the magnitude of the vectors. The pattern of transWSS was insensitive to inflow waveform; only unphysiologically high Womersley numbers produced substantial changes. However, transWSS was sensitive to changes in geometry. The curvature of the arch and proximal descending aorta were responsible for the principal features, the non-planar nature of the aorta produced asymmetries in the location and position of streaks of high transWSS, and taper determined the persistence of the streaks down the aorta. These results reflect the importance of the fluctuating strength of Dean vortices in generating transWSS.

## 1 Introduction

The non-uniform distribution of atherosclerosis within arteries is widely attributed to influences of haemodynamic wall shear stress (WSS). The low, oscillatory WSS theory (Caro et al., 1971, Ku et al., 1985) underlies most current investigations of lesion localisation but not all studies have supported it (Peiffer et al., 2013b). Mohamied et al. (2015) showed that the multidirectionality of WSS during the cardiac cycle, characterised by the transverse wall shear stress (transWSS) metric (Peiffer et al., 2013a), may be a more significant factor. Subsequent studies have shown that high transWSS is collocated with atherosclerotic plaque prevalence in minipigs (Pedrigi et al., 2015), and is associated with transitional flow instabilities in regions prone to wall remodeling (Bozzetto et al., 2015, Ene-Iordache et al., 2015). There is increasing interest in multidirectional near-wall flow, with alternative indices being proposed (Arzani and Shadden, 2016, Morbiducci et al., 2015, Gallo et al., 2016).

TransWSS is the average over the cardiac cycle of WSS components perpendicular to the temporal mean WSS vector, with which endothelial cells are assumed to align (Chien, 2007). This provides the biological hypothesis that endothelial cells may be adversely affected by cross-flow, and indeed direct *in vitro* evidence supports this view (Wang et al., 2013). High transWSS may arise from large fluctuations of a small shear vector, small fluctuations of a large shear vector, or small fluctuations of a modest shear vector over a larger portion of the cardiac cycle, to name but three examples. The nature of the arterial flows captured by transWSS and the sensitivity of transWSS to waveform and geometry are not understood. This study uses numerical methods to investigate the origins of transWSS in rabbit aortic geometries, in simplified versions of such geometries, and with modified inflow and outflow conditions. It focuses on large-scale features; flows around branch ostia will be considered in a later paper.

## 2 Methods

### 2.1 Geometries

Most simulations used geometries of immature rabbit thoracic aortas reconstructed from micro-CT scans of vascular corrosion casts (Peiffer et al., 2012).

Two idealised geometries, both lacking taper and branches, were constructed from the centerline of one of these realistic geometries using SolidWorks: torsion and curvature were preserved in one (“non-planar” case) and only curvature in the second (“planar” case). The centerline was obtained using VMTK (Antiga et al., 2003). Curvature, *κ*, is the deviation of the centerline from a straight line and torsion, *τ*, is the twisting of the plane of curvature; both were computed using MATLAB 2014a. For the curve ***r***(*s*), parametrised by the curvilinear abscissa *s*:

$$\kappa \left(s\right)=\;\frac{\left|r׳\left(s\right)\times r׳\left(s\right)\right|}{{\left|r׳\left(s\right)\right|}^{3}},$$

$$\tau \left(s\right)=\;\frac{\left[r\left(s\right)\times r׳\left(s\right)\right]\;∙\;r׳׳(s)}{{\left|r׳\left(s\right)\times r׳\left(s\right)\right|}^{2}}.$$

The Frenet–Serret formulae, which define the three unit vectors for the Frenet frame (tangent **T**, normal **N**, and binormal **B**), are:

dT/ds=κN,dN/ds=−κT+τB,dB/ds=−τN.

These, together with the definition of the tangent, T=dr/ds**,** allow a system of linear first-order differential equations to be solved for τ=0, which gives the centerline curve for the planar geometry.

### 2.2 Computational fluid dynamics

Time-dependent simulations were carried out on ~300 cores using the spectral/*hp* element framework Nektar++ (Karniadakis and Sherwin, 2013, Cantwell et al., 2015); two cycles were sufficient to dissipate initial transients. Rigid walls and Newtonian rheology were assumed with blood density ρ=1044 kg m^-3^ (Kenner et al., 1977) and dynamic viscosity μ=4.043 g m^-1^ s^-1^ (Windberger et al., 2003). Tetrahedral element meshes with a 0.02*D*-thick prismatic element boundary layer (where *D* is the aortic root diameter) were generated with STAR-CCM+ using curvature-based surface triangulation. Minimum and average element sizes were approximately 0.007*D* and 0.25*D*, respectively, giving ~150,000 elements. Meshes equivalent to approximately 50 M linear tetrahedral elements were obtained by using fifth-order polynomial expansions. Mesh convergence and *p*-convergence tests were carried out; spatially averaged WSS at peak systole differed by 0.01% using polynomial orders 3 and 5, and by 1% using meshes of 140,000 and 200,000 elements.

**Fig. 1:**
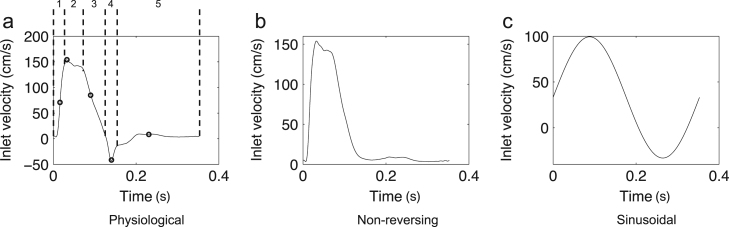
Aortic flow waveforms applied at the aortic root (inlet), and at branch outlets in cases where branch flow was modeled. Five phases of the cycle that were investigated further are delineated by dotted lines (a); the five time-points (numbered) chosen to represent each phase are indicated by the circles on the waveform.

A blunt inflow velocity profile was assumed at the aortic root and parabolic profiles at each branch outlet. These were initially modulated with a waveform (Fig. 1a) computed for the proximal descending aorta from a 1-D pulse wave propagation model (Alastruey et al., 2009). Further simulations were carried out with the reverse flow portion of the waveform removed (Fig. 1b) and with a sinusoidal waveform (Fig. 1c). A spatially- and temporally-averaged inflow Reynolds number of 300 was assumed.

Flow splits to the arch branches were based on experimental measurements (Barakat et al., 1997) with 14.7% to the brachiocephalic trunk and 7.1% to the left subclavian artery, and 2% of the descending aortic flow to the ten intercostal arteries (see Mohamied et al., 2015). Murray׳s law (Murray, 1926) was used for further flow divisions to individual branches. A no-slip condition was prescribed at the wall. A zero-velocity gradient boundary condition was imposed at the outlet, with a 0.75*D*-thick absorption layer (Israeli and Orszag, 1981) applied immediately upstream to damp out any instability introduced during the reverse flow portion of the cycle.

### 2.3 WSS parameters

Velocity gradients and consequent stress tensors were computed for all prismatic elements and projected on to the surface to obtain the WSS vector, ***τ***_***w***_. Time-averaged WSS (TAWSS) and transWSS were computed. Instantaneous values at the *i*th time-point can also be computed; they were defined as:

$$\mathrm{WSS}=\left|\tau_{w,i}\right|,\;\mathrm{transWSS\_i}=\tau_{w,i}\;∙\;\left(n\times \frac{\int_{0}^{T}\tau_{w}\mathrm{dt}}{\left|\int_{0}^{T}\tau_{w}\mathrm{dt}\right|}\right)\;,$$

where ***n*** represents the surface normal.

A novel parameter, named the cross-flow index (CFI), was also computed. The instantaneous value (CFI_i) at the *i*th time-point was defined as:

$${\mathrm{CFI}}_{i}=\;\frac{{\mathrm{transWSS}}_{i}}{\mathrm{WSS}}\;=\;\frac{\tau_{w,i}}{\left|\tau_{w,i}\right|}\;∙\;\left(n\times \frac{\int_{0}^{T}\tau_{w}\mathrm{dt}}{\left|\int_{0}^{T}\tau_{w}\mathrm{dt}\right|}\right)\;,$$

and represents the directionality of WSS with no account taken of magnitude. It is the sine of the angle between the temporal mean WSS vector and the instantaneous WSS vector, and differs from the aneurysm formation index (Mantha et al., 2006), which is the cosine of this angle. The CFI is the time-average of these instantaneous components defined as:

$$\mathrm{CFI}=\;\frac{1}{T}\int_{0}^{T}\frac{\tau_{w}}{\left|\tau_{w}\right|}\;∙\;\left(n\times \frac{\int_{0}^{T}\tau_{w}\mathrm{dt}}{\left|\int_{0}^{T}\tau_{w}\mathrm{dt}\right|}\right)\mathrm{dt}\;.$$

Computed shear distributions were mapped onto a rectangular parametric space using VMTK.

## 3 Results

### 3.1 Time-averaged physiological WSS metrics

**Fig. 2:**
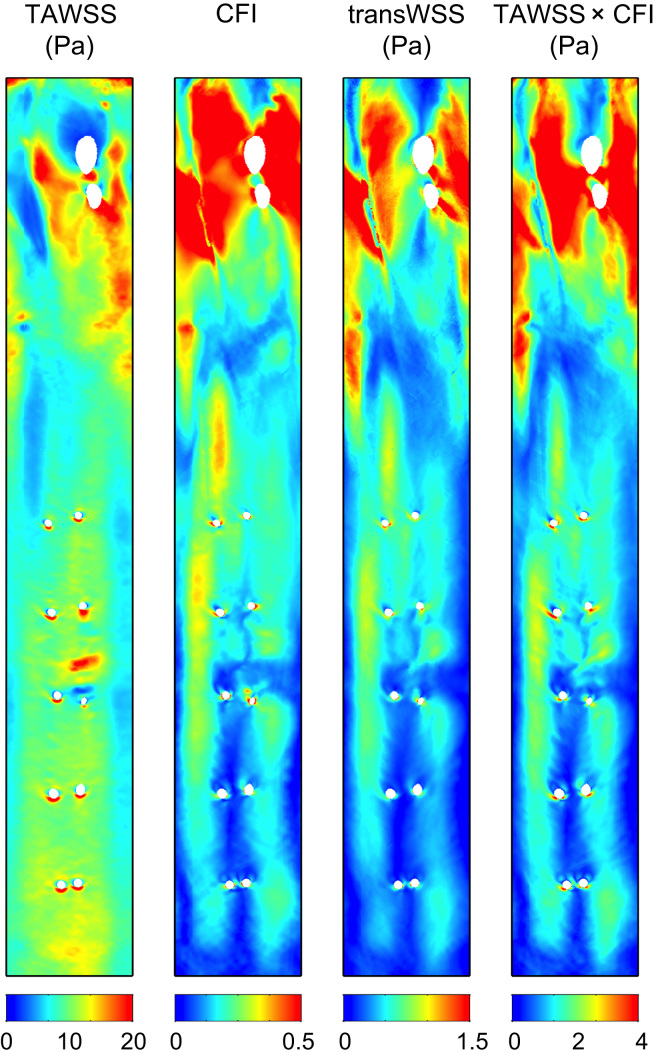
Distributions of WSS metrics on the unwrapped luminal surface, viewed *en face*, for the anatomically and physiologically realistic simulation. TAWSS, transWSS and the product of time-averaged WSS and CFI are given in Pascals. Mean flow is from top to bottom, from the aortic root, over the arch (with two branch holes) and past the 5^th^ intercostal pair. Anatomical left and right correspond to the right and left of each map, respectively. Note the different colour bars.

Time-averaged metrics for an anatomically realistic geometry with physiological boundary conditions are shown in Fig. 2. The maps of TAWSS and CFI bear little resemblance to each other - hence highly multidirectional flow was not consistently associated with either a high or a low mean shear stress. The map of transWSS more closely resembles the map of CFI than that of TAWSS; differences between them are evident chiefly in the segment between the branches in the arch and the first intercostal branches but even there they are subtle.

Since instantaneous transWSS is equal to WSS × CFI_i, the product of TAWSS and CFI is also shown in Fig. 2. It resembles the CFI map in the descending segment: there are few places where a relatively low or high TAWSS appears to cause a noticeable decrease or increase in TAWSS × CFI. Proximal to the first branch in the arch and on the right lateral wall proximal to the first intercostal ostia, however, the pattern of TAWSS × CFI does show influences of the TAWSS. The pattern of TAWSS × CFI is also similar to that of transWSS, but there are discrepancies between them in the same regions, and, most strikingly, the values of TAWSS × CFI are approximately twice as high as the values of transWSS.

These complex interrelations between the time-averaged metrics indicate that temporal changes in WSS and CFI_i during the cardiac cycle must be important determinants of transWSS; to understand the build up of transWSS, instantaneous distributions must be analysed.

### 3.2 Different determinants of instantaneous transWSS patterns

**Fig. 3:**
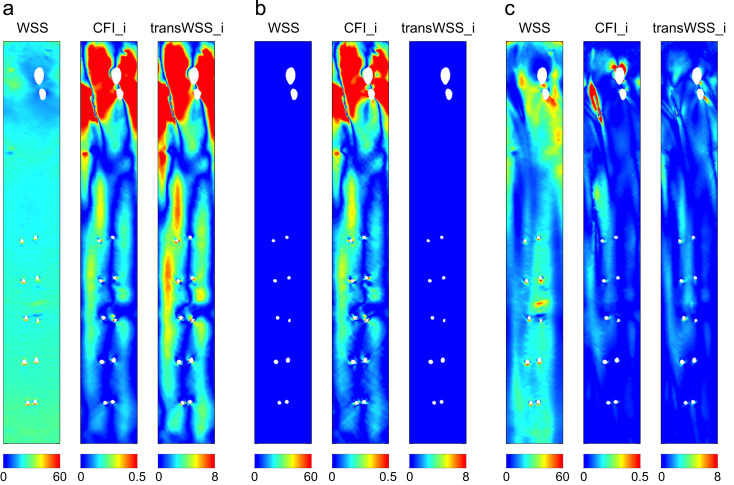
Maps of instantaneous WSS, CFI and transWSS for the physiological case, demonstrating the decomposition of the instantaneous transWSS vector into its magnitude (WSS) and direction (CFI_i) components. Three points in the cardiac cycle (a-c) represent three scenarios: (a) where WSS is spatially uniform and non-zero, (b) where WSS is near 0, and (c) where WSS is non-zero and varies spatially. WSS and transWSS_i are in Pascals.

Fig. 3a–c show WSS, CFI_i and transWSS_i at three instants in the cardiac cycle, chosen to illustrate contrasting scenarios. In Fig. 3a, the WSS pattern is spatially uniform and non-zero, and it acts as a scaling function to the spatially varying CFI_i, with the pattern of transWSS_i therefore resembling that of the CFI_i (scenario a). In Fig. 3b, WSS is near-zero, which precludes any pattern of transWSS_i even though CFI_i has high and variable values (scenario b). In Fig. 3c, where WSS is non-zero and spatially varying, the distribution of transWSS_i is not merely a scaled version of the CFI_i pattern but a modulated version; this is particularly noticeable in the arch (scenario c).

### 3.3 Components and values of transWSS_i over the cardiac cycle

**Fig. 4:**
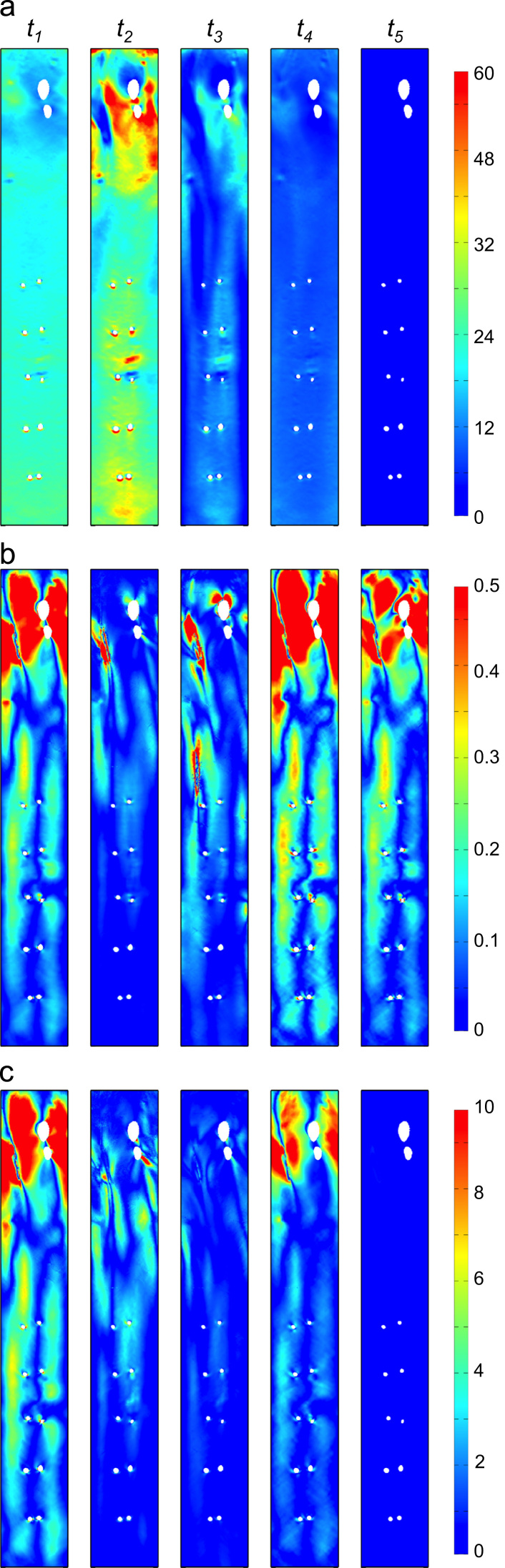
Distribution of instantaneous WSS (a), CFI (b) and transWSS (c) at five times in the cardiac cycle (columns *t*_*1*_–*t*_*5*_) for the physiological case. The five time-points are defined in Fig. 1a. WSS and transWSS_i are in Pascals.

We next consider how these different scenarios contributed to the transWSS averaged over the cardiac cycle. Five distinctive phases were observed. During the acceleration phase of systole (phase 1 in Fig. 1a, column *t*_*1*_ in Fig. 4), WSS was spatially uniform and high, and CFI_i showed some of its highest values as, consequently, did transWSS_i, and with a similar pattern. As the acceleration continued, WSS increased, CFI_i decreased, and transWSS_i values remained reasonably constant (not shown).

At peak systole (phase 2 in Fig. 1a, column *t*_*2*_ in Fig. 4), WSS attained its maximal values and CFI_i was at its lowest. TransWSS_i values decreased compared to phase 1. All three metrics remained reasonably unchanged over the systolic plateau (not shown). As the flow began to decelerate towards zero (phase 3 in Fig. 1a, column *t*_*3*_ in Fig. 4), CFI_i steadily increased but WSS decreased more, relative to phase 2, so transWSS_i values were lowered.

As the flow reversed (phase 4 in Fig. 1a, column *t*_*4*_ in Fig. 4), WSS was still non-zero (although lower than before) and uniform, while CFI_i was maximal; in combination, this gave transWSS_i values approaching those seen in phase 1. Finally, the nearly stagnant flow during diastole (phase 5 in Fig. 1a, column *t*_*5*_ in Fig. 4) gave near-zero WSS values and hence negligible transWSS_i, despite high CFI_i values.

Of the five phases, phases 1, 3 and 4 developed transWSS_i values according to scenario (a) described in Fig. 3. Peak systole (phase 2) was the only part where scenario (c) dominated. Lastly, scenario (b) dominated during the long diastolic part (phase 5).

### 3.4 Temporal mean WSS vector

**Fig. 5:**
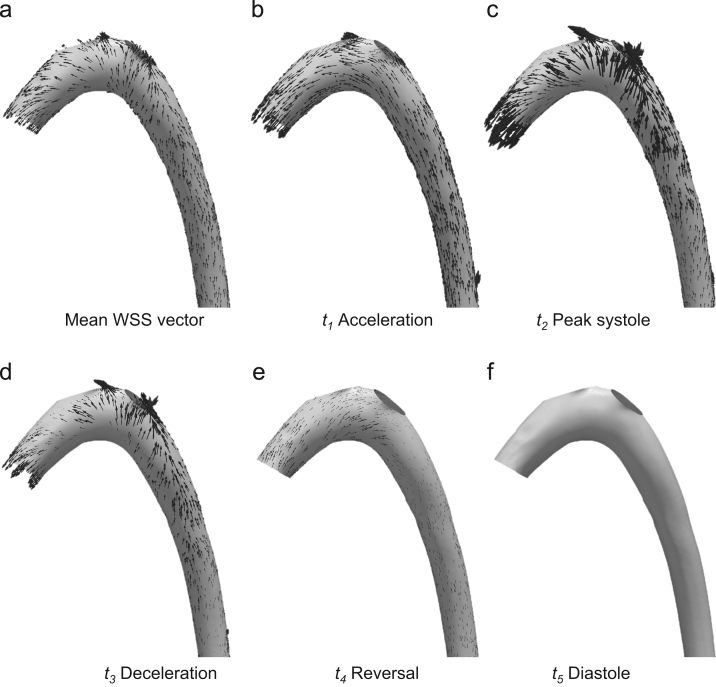
WSS vectors over the arch for the physiological case. (a) Temporal mean WSS vectors, (b)–(f) instantaneous WSS vectors for the five time-points shown in Fig. 1a. Note that arrows point in the reverse direction to the flow.

The influence of the five phases on the temporal mean WSS vector is assessed next; Fig. 5 shows the temporal mean WSS vectors (Fig. 5a) and the instantaneous WSS vectors (Fig. 5b-f) for each phase, focusing on the arch as an example. The helical vectors during peak systole were the strongest over the cycle and most influenced the temporal mean vector. Although the vectors reversed direction in late systole/early diastole, their strengths were not sufficient to compete with those during phases 1–3. Shear vectors had negligible magnitudes in diastole.

### 3.5 Influence of aortic inflow waveform

**Fig. 6:**
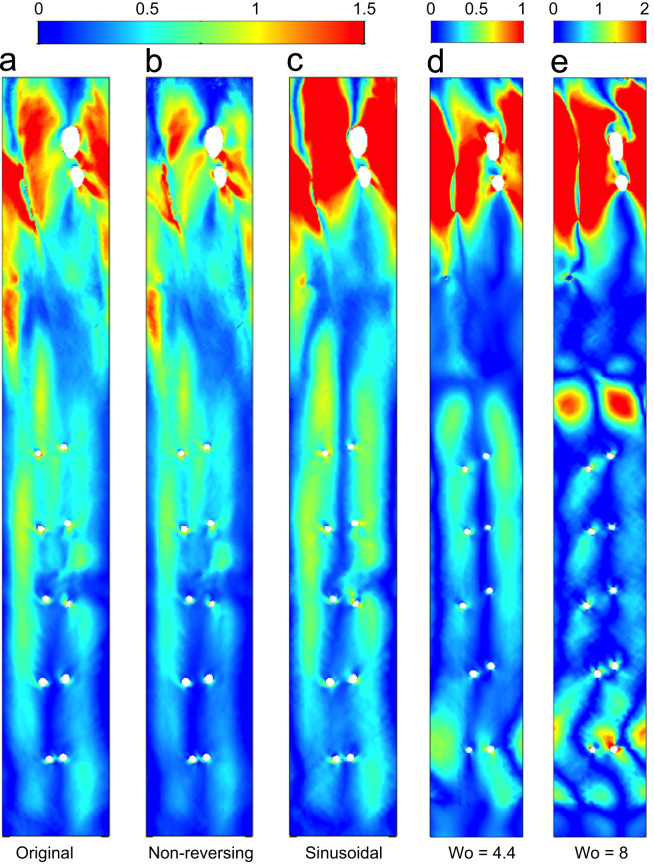
Distributions of transWSS (Pa) for different inflow waveforms: (a) the physiological case, (b) the physiological case with reverse flow removed and (c) a sinusoidal waveform. (d) and (e) give transWSS for physiological waveforms at a physiological Womersely number of 4.4 and a non-physiological value of 8, respectively. (These two simulations were run in a geometry from a different immature rabbit.)

Removing the reverse flow portion of the waveform had little effect on the magnitude or distribution of transWSS in the descending aorta; in the arch, the magnitude was reduced but the pattern was almost unchanged (Fig. 6a vs b).

The sinusoidal case (Fig. 6c) was different to the physiological case. Values in the arch were increased and there was accentuation of the central and left-hand longitudinal streaks seen proximal to the first intercostal pair and extending to the second pair. Nevertheless, the overall distribution of transWSS still resembled the physiological case.

Comparing cases with physiological and non-physiologically high Womersley numbers (Fig. 6d vs 6e), the longitudinal streaks fragmented and magnitudes in the arch and descending aorta increased at the higher value.

### 3.6 Influence of outflow and geometry

**Fig. 7:**
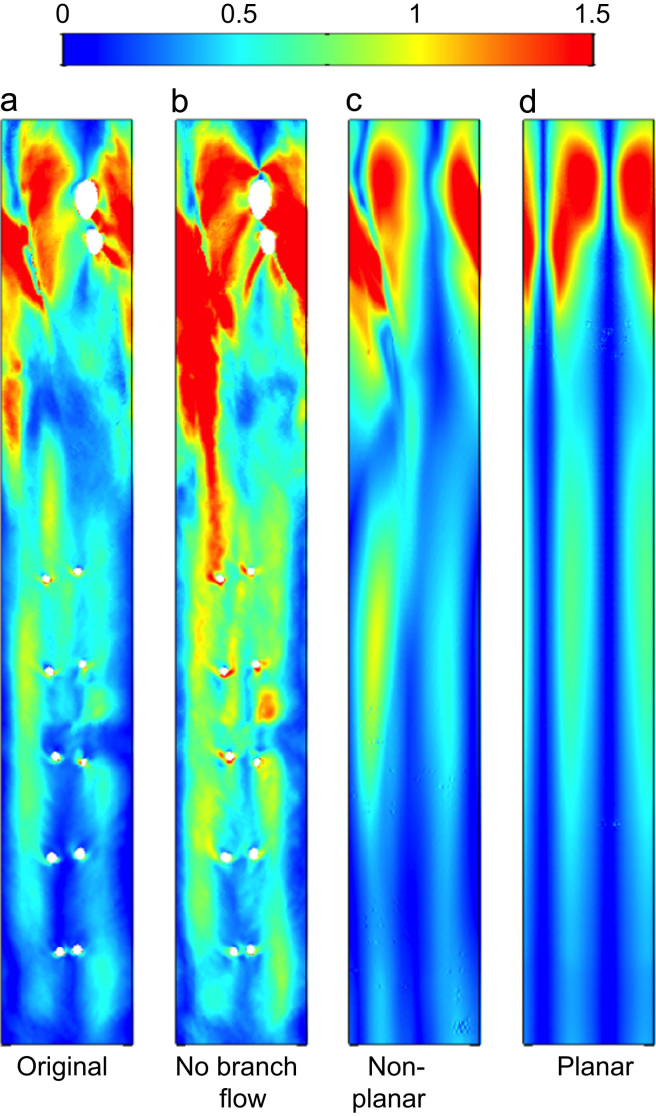
Distributions of transWSS (Pa) for: (a) the original anatomically-realistic geometry, (b) the original geometry with flow down all branches removed for better comparison with the idealised, branch-less cases, (c) an idealised non-tapered case with torsion and curvature of the original geometry preserved (“non-planar”) and (d) an idealised non-tapered case with torsion removed and only curvature preserved (“planar”).

To provide a baseline against which to compare simulations in idealised geometries without branches, the physiological case (Fig. 7a) was run with no outflow to branches (Fig. 7b). This increased the magnitude of transWSS at locations from the first branch onwards, as expected. The large-scale pattern remained fairly consistent, but there was a disproportionate increase in the inner curvature of the arch; presumably flow into the branches of the outer curvature produced a particularly slow near-wall flow in this region.

In the anatomically realistic geometry, the hydraulic diameter increased from the root to the arch and then decreased at the start of the descending aorta, eventually becoming smaller than at the root; it remained constant thereafter. Removing taper (Fig. 7c) reduced transWSS values in the distal arch and descending aorta.

In the planar case (Fig. 7d), two well-defined symmetrical streaks of high transWSS developed in the arch and continued downstream; they were strengthened in the descending aorta. The asymmetry in the location and strengths of the streaks in the descending segment seen in all previous cases was eliminated. Interestingly, the streaks still weakened at the distal end of the arch, and then became stronger once more, suggesting that local curvature in the descending aorta is an important factor.

## 4 Discussion

TransWSS is the average over the cardiac cycle of the instantaneous product of CFI_i and WSS. The present study demonstrated that although transWSS had a similar pattern to TAWSS × CFI (i.e. to the product of the two cycle-averaged metrics) there were discrepancies and, strikingly, it had only half the magnitude, implying that it is important to examine the instantaneous interactions of CFI_i and WSS. The largest values of instantaneous transWSS (transWSS_i) arose during acceleration towards peak systole and during flow reversal. However, both these periods are of short duration and the build-up over the longer peak systolic and deceleration periods gave comparable contributions to transWSS, despite transWSS_i having smaller magnitudes.

At those parts of the cycle where transWSS_i built up, the spatial distribution of transWSS was predominantly determined by the spatial distribution of CFI_i because WSS was relatively uniform. Note also that CFI_i was at its lowest in parts of the cycle where the WSS was highest; that is because CFI_i is defined by the deviation of the instantaneous WSS vector from the cycle-averaged WSS vector and it is those portions that most define the mean WSS vector, and thus where the instantaneous WSS vector deviates least from it.

Negligible differences in the pattern of transWSS were seen on removing the reverse flow portion of the waveform, although magnitudes were reduced. This portion of the cycle does not significantly influence the pattern because its duration is short and because it generates the same distribution of transWSS as the acceleration phase (compare columns t1 and t4 in Fig. 4). Sloop et al., 1998 showed in human aortas that flow reversal is present in young adults but not in older subjects. The present study suggests that this change may not have a significant effect on transWSS, at least at the large scale.

**Fig. 8:**
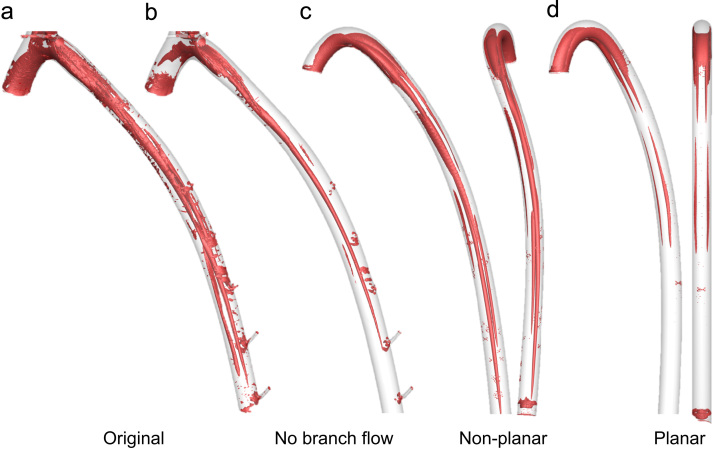
Isocontours of the Q-criterion showing vortical structures at time-point *t*_*3*_ (defined in Fig. 1a) for the geometrical cases described in Fig. 7. Two viewpoints are shown for the idealised “non-planar” and “planar” cases.

Even for a sinusoidal waveform, the fundamental pattern of transWSS remained unchanged. Only the use of a high Womersely number disrupted the streaks normally present in the descending aorta. This effect can be understood if we consider an alternative dimensionless parameter, the reduced velocity *U*_*red*_, which represents the length in aortic diameters that the mean velocity travels during one cycle. For Wo = 4.4 and 8 (~ 250 and 840 heart beats per min), *U*_*red*_ = 15.4 and 4.6 respectively, i.e. at the higher Wo, flow structures generated during one cycle do not propagate far before the next cycle begins, leading to interaction of vortical structures and, crucially for transWSS, more complex and different flow behavior. The effect of Wo on large-scale flow structures and near-wall vorticity (but not transWSS) has been addressed previously (Pitt, 2006, Doorly and Sherwin, 2009). An example of such instantaneous vortical structures in the anatomically realistic geometry is shown in Fig. 8a, by means of isocontours of the Q-criterion (Hunt et al., 1988), and gives a broad qualitative characterisation of the flow.

The influence that vortical structures have on transWSS is most evident when we consider the sensitivity of the pattern of transWSS to changes in geometry. In the idealised planar geometry, where taper and torsion were removed, the two streaks of high transWSS (Fig. 7d) appear to reflect a Dean vortex pair that develop due to the primary curvature of the arch and which are reinforced by local curvature in the proximal descending segment (Fig. 8d).

When torsion was added to the idealised geometry, the streaks of high transWSS in the arch became disconnected from the two in the descending aorta (Fig. 7c), suggesting that they arise from two different primary curvatures, one in the arch and one in the distal segment. The steaks in the descending aorta also became asymmetric in magnitude and shifted towards the anatomical right of the map. Non-planarity broke the symmetry of Dean vortex pairs (Fig. 8c), as previously described (Caro et al., 1996, Lee et al., 2008, Sherwin et al., 2000). Vortices may be reinforced by merging with the wall vorticity layer, or annihilated if encircled by opposing-valued wall vorticity (Doorly and Sherwin, 2009, Zabielski and Mestel, 1998). The evolution of the vortex pair for the non-planar case explains the different transWSS patterns obtained.

Finally, the presence of taper did not alter fundamental patterns of transWSS (Fig. 7b) but accentuated the magnitude of the streaks in the descending aorta, presumably due to increased WSS and, as previously speculated (Peiffer et al., 2013a), the extension of the vortices.

The Dean number (De) is directly proportional to the bulk axial velocity, which peaks in systole. (At maximum curvature in the aortic arch, De averaged over the cycle was 350, whereas at peak systole it was 1400). The Dean vortex pair is consequently at its strongest and it is the flow structures during this portion of the cycle that dominate the mean WSS vector (Fig. 5c). Streaks of high transWSS arise not from any lateral motion of the vortical structures but simply due to variation in the near-wall secondary flow (and secondary vorticity) that results from the vortex pair growing and decaying over the cycle.

The present study has a number of limitations. The simulations assumed rigid walls and Newtonian rheology but these conventional approximations have only small influences on models of flow in large arteries (Perktold and Rappitsch, 1995, Lee and Steinman, 2007) and are unlikely to have altered the effects of changing waveform or geometry. A potentially more serious limitation arises out of the finding that geometry is crucial to the global pattern of transWSS. The vascular corrosion casts of the rabbit aorta used in this study were obtained by placing the rabbit flat on its back, an unnatural posture that may introduce artificial extension of the spine. In human contortionists extension angles of up to 8° are observed for the first few thoracic vertebrae, and 3° for intermediate ones (Peoples et al., 2008), so there is a possibility that the supine posture influences the geometry obtained for the aorta even within the thoracic region. To capture the patterns and values of transWSS that occur *in vivo* on the time scale over which endothelial cells align to flow, a characterisation of the average geometry would be preferable.

## 5 Conclusion

Without the pulsatile nature of blood flow there would be no variation in near-wall velocity with time and endothelial cells would never experience cross-flow. Pulsatility is clearly necessary for the existence of multi-directionality, yet the precise nature of the aortic waveform does not dominantly influence the pattern of transWSS. Instead, the pattern is primarily dependent on geometry. In particular, it is the curvature of the arch and the proximal descending aorta that is responsible for the global features of transWSS, the non-planar nature of the aorta that is responsible for the differences between the two dominant streaks and for the more complex patterns seen *in vivo*, and taper that determines the persistence of these effects down the aorta.

Finally we note that investigation of the relation between transWSS and disease has so far been limited to early fatty streaks. Raised atherosclerotic lesions, aneurysms and dilatation involve substantial alteration of vessel geometry, whilst valvular disease can be associated with prolonged periods of enhanced reverse flow, and it may therefore also be of interest to study the role of transWSS in advanced disease.

## Conflict of interest statement

The authors declare no conflict of interest.
